# Hearing Results, Quality of Life, Patient Satisfaction, and Postoperative Complications of Day-case Versus Inpatient Stapes Surgery for Otosclerosis in Adults: A Randomized Controlled Trial

**DOI:** 10.1097/ONO.0000000000000019

**Published:** 2022-10-27

**Authors:** Laura S. M. Derks, Isabelle Borgstein, Digna M. A. Kamalski, Hans G. X. M. Thomeer, Rinze A. Tange, Wilko Grolman, Robert J. Stokroos, Inge Wegner

**Affiliations:** 1 Department of Otorhinolaryngology—Head and Neck Surgery, University Medical Center Utrecht, Utrecht, the Netherlands; 2 Brain Center Rudolf Magnus, University Medical Center Utrecht, Utrecht, the Netherlands; 3 Causse Ear Clinic, Traverse de Béziers, Colombiers, France; 4 Department of Otorhinolaryngology—Head and Neck Surgery, University Medical Center Groningen, Groningen, the Netherlands.

**Keywords:** Audiometry, Day-case, Hearing results, Inpatient, Otosclerosis, Patient satisfaction, Quality of life, Readmission, Stapedotomy, Stapes surgery, Tinnitus, Vertigo

## Abstract

**Objective:**

To investigate the effect of day-case stapes surgery on hearing results, quality of life, patient satisfaction, and complications rates, compared with inpatient stapes surgery.

**Study Design:**

A single-center, nonblinded, randomized controlled trial in a tertiary referral center.

**Methods:**

One hundred twelve adult patients planned for primary or revision stapes surgery were randomly assigned to either the day-case or inpatient treatment group. The effect on hearing outcomes (primarily), hearing benefits, quality of life, patient satisfaction, postoperative complications, and causes of crossover or readmission (secondarily) were assessed using auditory evaluations, questionnaires, and patients’ charts over a follow-up period of 1 year.

**Results:**

Audiometric measurements and postoperative success rates were not different between the inpatient and day-case group. There were no statistically significant differences between both groups regarding the overall quality of life (QoL) (HUI3), disease-specific QoL (GHSI), change in postoperative health status (GBI), and postoperative complications rate. We found a high patient satisfaction toward the day-case approach. Six patients allocated to the inpatient group requested same-day discharge. Of the day-case patients, there was a crossover rate to inpatient care of 38% (20 patients), mainly due to postoperative nausea and vomiting (25%), vertigo (20%), or dizziness (40%).

**Conclusion:**

We found no significant differences in outcomes of audiometric measurements, QoL, patient satisfaction and postoperative complications following day-case, and inpatient stapes surgery. Therefore, stapes surgery in a day-case setting seems to be a feasible approach in terms of postoperative outcome, safety, and desirability when taking patient selection and surgical planning into account. Besides this, the familiarity with a day-case approach of both patient and the surgical team, will increase the acceptance and feasibility of day-case stapes surgery.

## INTRODUCTION

Otosclerosis is a disease characterized by abnormal sponge-like bone growth in the otic capsule, causing progressive hearing loss, vertigo, or tinnitus and can be treated surgically by performing stapes surgery (1,2). Stapes surgery has been proven to be a safe and effective treatment option for otosclerosis with low complication rates (3). Permanent sensorineural hearing loss (SNHL) is the most dreaded complication and has been reported to be less than 2% in large series following primary stapes surgery and 1% to 8% in revision stapes surgery (4–14). Other complications associated with stapes surgery are postoperative vertigo (1%–26%), tinnitus (2%–54%), temporary taste alteration due to manipulation of the chorda tympani nerve (2%–16%), perforation of the tympanic membrane, and facial nerve palsy (<1%) (6,15–24).

Many otologic procedures that involved overnight hospital stay in the past are presently being performed on an outpatient basis due to several reasons (25–29). Day-case surgery is associated with shorter waiting time for surgery and reduced risk of hospital-acquired infections (30). The increased feasibility of performing surgery in a day-case setting could also be a result of anesthetic improvements, including local infiltration of analgesics, the use of less and shorter-acting intraoperative anesthetic drugs, especially morphine, and inhalants, and better prophylaxis for postoperative nausea and vomiting (PONV) (31,32). Ear surgery in general and stapes surgery in particular is well suited to a day-case approach. The population tends to be younger with less comorbidity than in many other specialties and procedures are associated with well-documented, low complication rates. At the start of this trial in 2014 in our clinic, stapes surgery involved overnight hospital stay.

Literature comparing day-case and inpatient surgery supporting this statement is scarce. Therefore, the aim of this randomized controlled trial is to investigate the effect of day-case stapes surgery on hearing results, QoL, patient satisfaction, and postoperative complications, compared with inpatient stapes surgery.

## MATERIALS AND METHODS

This article is based on data acquired in a single-center, nonblinded, randomized controlled trial, approved by the Institutional Review Board of the University Medical Center Utrecht (NL45219.041.13). This study was registered in the Netherlands Trial Register (www.trialregister.nl; NTR4133, August 21, 2013). The complete study protocol was published in February 2016 (33). There were no changes to the study methods or outcomes after the study commenced. The data are reported according to the CONSORT Statement (34–36).

### Study Population

**FIG. 1.**
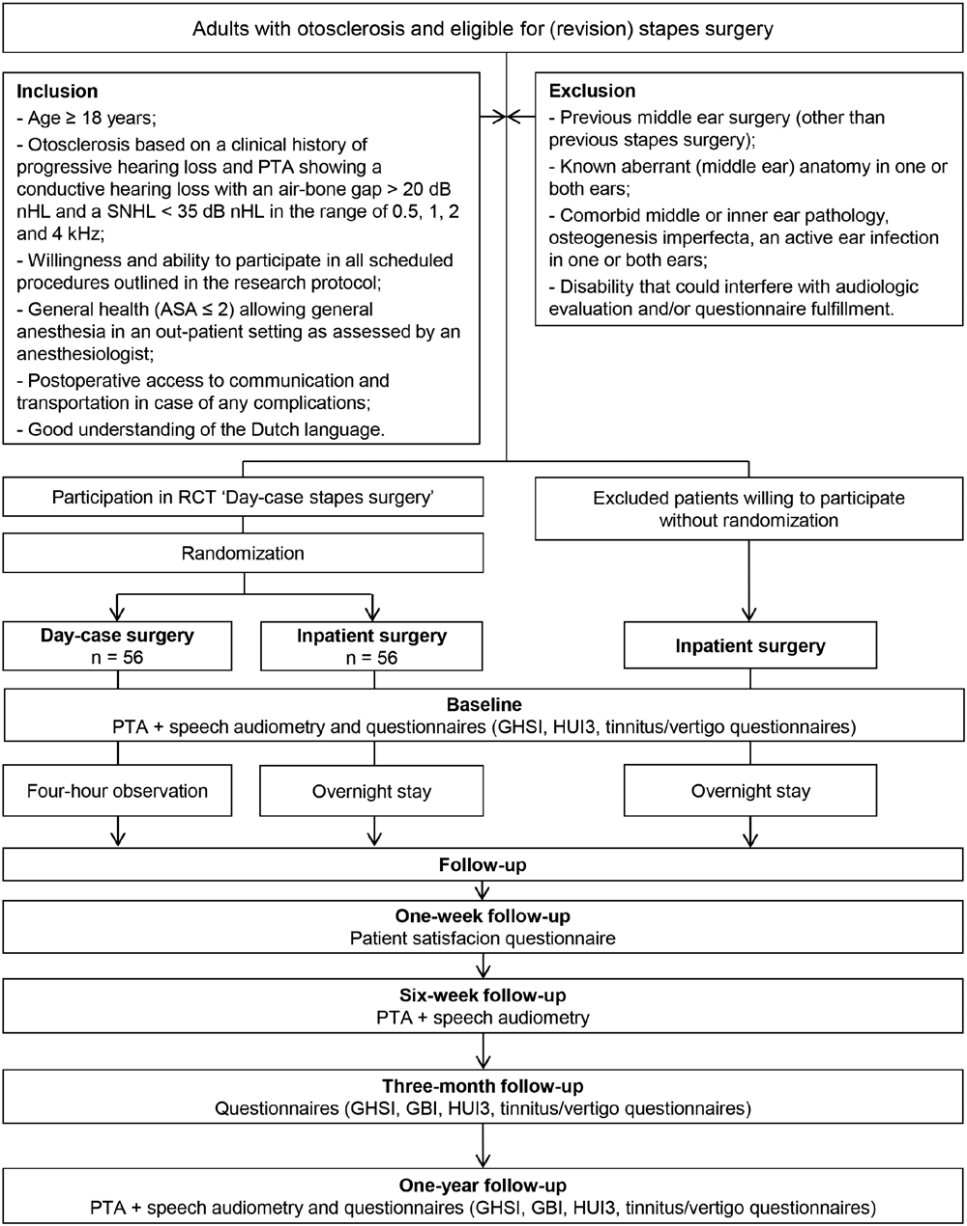
Flow chart of day-case stapes surgery study. dB indicates decibel; GBI, Glasgow Benefit Inventory; GHSI, Glasgow Health Status Inventory; HUI3, Health Utilities Index—Mark 3; n, number of patients; PTA, pure-tone audiometry; SNHL, sensorineural hearing loss.

Adult patients undergoing primary or revision stapes surgery were eligible to participate if they met all inclusion criteria (Fig. 1).

### Sample Size Calculation

To detect a clinically relevant difference in postoperative mean air-conduction (AC) of 5 dB with a standard deviation (SD) of 10 between the day-case and the inpatient group with an alpha of 0.05 and a power of 80%, 51 patients per group were needed. To anticipate 10% withdrawal of patients, a total of 56 patients were recruited per group.

### Randomization, Blinding, and Treatment Allocation

Patients were enrolled by one of two researchers (authors LD and IW). After receiving informed consent, patients were allocated to either the conventional group (inpatient surgery) or the day-case group using a web-based randomization program (Julius Center, UMC Utrecht, Utrecht, the Netherlands). Patients were randomly allocated into two groups with stratification for age. Block randomization was used with an allocation ratio 1:1. The randomization chart, including block size, was established by an independent data manager before the start of the study. Consequently, treatment allocation sequence was concealed for patients, care providers, and researchers. Blinding of the involved was not possible because patients and care providers would be aware of the surgical setting and hospital stay. Crossover was defined as admission of a day-case patient (i.e., admission) or discharge on the day of surgery of patients allocated to the inpatient group. Readmission was defined as admission after initial discharge. In case of crossover, patients were asked to complete their follow-up, and analyses were carried out on an intention-to-treat basis.

### Baseline

Baseline characteristics were assessed using patients’ charts. Preoperatively, pure-tone audiometry and speech audiometry were performed. Furthermore, patients were asked to fulfill the Health Utilities Index—Mark 3 (HUI3) (37,38) and Glasgow Health Status Inventory (GHSI) (39) questionnaires as a baseline measurement. If patients suffered from tinnitus and vertigo preoperatively, they were asked to complete tinnitus and vertigo questionnaires. These included the Tinnitus Handicap Index (THI) (40,41), Tinnitus Questionnaire (TQ) (42,43), Dizziness Handicap Inventory (DHI) (44,45), and the Utrecht Burden Questionnaire for tinnitus (UBQT) and vertigo (UBQV) (Appendix 1, http://links.lww.com/ONO/A6).

### Intervention

All surgical procedures were performed under general anesthesia and by four surgeons in the same tertiary referral center (University Medical Center Utrecht). Patients allocated to the inpatient group were admitted one day before or on the day of surgery and were discharged one day after surgery. Patients allocated to the day-case group were admitted one day before or on the day of surgery and were discharged the day of surgery. If postoperatively patients were not physically capable of same-day discharge (e.g., due to PONV or dizziness) or if the surgeon did not support same-day discharge, patients would stay overnight.

### Primary Outcome Measures

The primary outcome was the postoperative mean air-conduction (AC) assessed at approximately 2 months and 1 year postoperatively. AC thresholds at 500, 1000, 2000, and 4000 Hz were measured and averaged. This was in accordance with the Committee on Hearing and Equilibrium guidelines for the evaluation of results of treatment of conductive hearing loss, except for substituting thresholds at 3000 Hz with those at 4000 Hz (46,47).

### Secondary Outcome Measures

The secondary outcome measures included additional audiometric measurements, subjective hearing benefits, QoL, patient satisfaction, postoperative complications (including tinnitus and vertigo), and causes of crossover and readmission.

Additional audiometric measures included additional pure-tone audiometric measurements and speech audiometry at approximately 2 months and 1 year postoperatively. Audiometric evaluation included mean postoperative bone-conduction (BC) thresholds and air-bone gap (ABG). AC and BC thresholds at 500, 1000, 2000, and 4000 Hz were measured and averaged. Mean ABG was defined as the difference between mean AC and BC thresholds. The rate of ABG closure to 10 dB or less and to 20 dB or less in both groups were calculated. SNHL was evaluated using frequencies 1000, 2000, and 4000 Hz and was defined as a deterioration in mean BC thresholds exceeding 10 dB. Speech discrimination scores were measured using a standard set of monosyllabic words and recording the correct word score in percentages. Furthermore, the speech recognition percentage at 65 dB (conversation level) (SRT) and the maximum achievable percentage score and corresponding dB score was determined (dB_max_).

Subjective hearing benefit was evaluated at 3 months and 1 year postoperatively using the GHSI and the Glasgow Benefit Inventory (GBI). Both questionnaires contain 18 questions assessing the effect of an otologic problem (GHSI) or of an otologic procedure (GBI) on QoL. The GHSI is scored into a total score and three subscores (general, social support, and physical health), all with a range from 0 (low health status) to +100 (high health status) (39). The GBI scores a total score and the same three domains as the GHSI, ranging from –100 (maximum negative benefit), through 0 (no benefit), to +100 (maximum benefit) (48,49).

General QoL was measured at 3 months and 1 year postoperatively by the HUI3. The HUI3 is a 15-item questionnaire measuring the general health by evaluating eight domains (vision, hearing, speech, ambulation, dexterity, cognition, emotion, and pain). The outcome is a multiattribute health status resulting in a utility score between –0.36 (a state worse than death) and +1.00 (perfect health) (37,38). We reported the mean multiattribute score and the hearing multiutility score.

The Utrecht Patient Satisfaction Survey was used to evaluate patient satisfaction regarding the day-case approach or hospital admission at one week postoperatively (Appendix 2, http://links.lww.com/ONO/A7).

The occurrence of postoperative complication, crossover, and readmission were prospectively registered in the patients’ chart. Complications were considered major if hospitalization or additional or revision surgery were required and minor if they resolved spontaneously or if only medication was required. Differentiation was made between perioperative (during surgery), directly postoperative, early (within 1 month after surgery), and late (within 1 year after surgery) complications. The presence and burden of tinnitus and vertigo were assessed at 3 months and 1 year postoperatively using tinnitus (THI, TQ, UBQT) and vertigo (DHI, UBQV) questionnaires. The UBQT and UBQV were also administered directly postoperatively in case of direct postoperative tinnitus and vertigo.

### Statistical Analyses

Means, SDs, and percentages were calculated. Differences in the baseline were analyzed using the independent-samples students *t*-test for continuous variables and the Fisher’s exact test for categorical variables. Normality was analyzed using mean, median, histogram, and boxplots. Nonparametric tests were used when the outcome data were not normally distributed. A *P* value <0.05 (2-tailed) was considered statistically significant.

For analysis of between-group differences in both primary and secondary outcomes, the independent-samples students *t*-test or nonparametric Mann-Whitney *U* test were used for continuous outcomes and the Fisher’s exact test for categorical outcomes. Again, a *P* value <0.05 (2-tailed) was considered statistically significant. Between-group mean differences, rate differences, and rate ratios with 95% confidence intervals (CIs) were calculated.

Missing values were handled using multiple imputation. Questionnaires that were returned empty were not imputed. All analyses were performed on an intention-to-treat basis, using SPSS version 25 (SPSS Inc., Chicago, IL).

## RESULTS

### Patient Flow

**FIG. 2.**
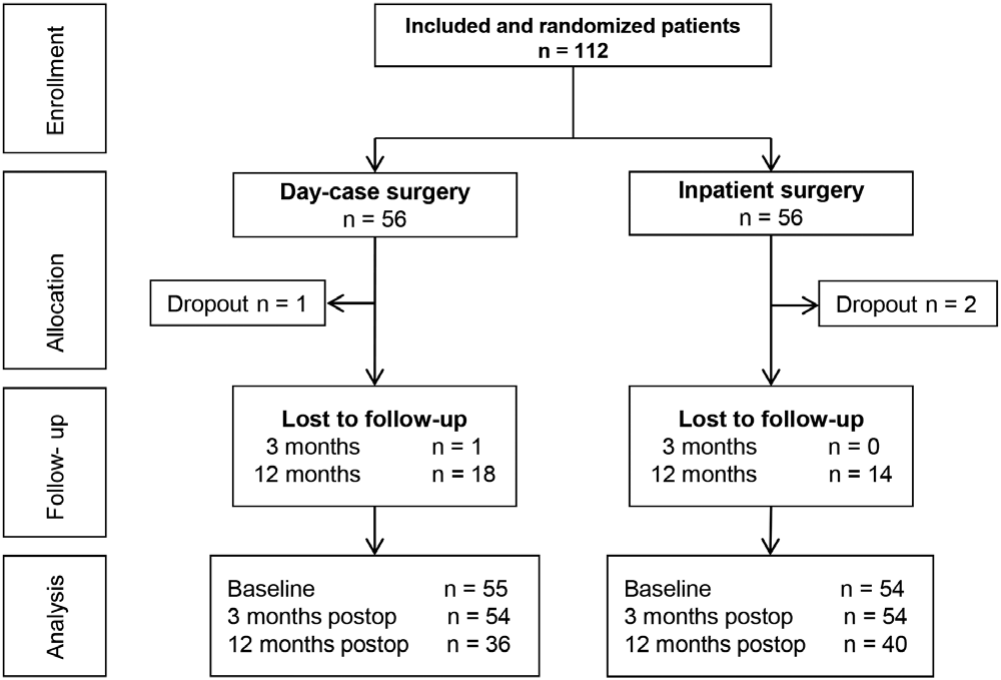
Flow chart of included patients. n indicates number of patients.

A total of 103 patients, planned to undergo 112 consecutive (revision) stapes surgeries, were included from April 2014 to December 2019 (Fig. 2). Follow-up took place from April 2014 to March 2021. Three patients dropped out after treatment allocation and before receiving treatment: two in the inpatient and one in the day-case group. Reasons for withdrawal were as follows: at patient’s request due to personal reasons (n = 2) and cancelation of the surgery due to pregnancy (n = 1, inpatient group). The three dropouts were excluded from all analyses, resulting in a total of 109 cases (100 patients). These 109 cases were analyzed for the primary outcome postoperative mean AC threshold at approximately 2 months and 1 year (n = 55 day-case, n = 54 inpatient).

### Baseline Characteristics

There were no significant between-group differences in the baseline characteristics (Table 1).

**TABLE 1.** Baseline characteristics

	Inpatient (n = 54)	Day case (n = 55)	Difference (95% CI)
Sex (male:female)	20:34	23:32	^a^
Age at surgery (mean [SD] in years)	47 (10)	48 (10)	–1 (–4 to 3)^b^
Family history (n, %)			
Hearing loss	22 (41)	25 (45)	^a^
Otosclerosis	12 (22)	13 (24)	
Negative for hearing loss/otosclerosis	15 (28)	16 (29)	
Unknown	5 (9)	1 (2)	
Preoperative hearing evaluation, operated side			
AC (mean, SD) dB^c^	52 (10)	53 (12)	0 (–4 to 4)^b^
BC (mean, SD) dB^c^	19 (6)	21 (7)	–2 (–5 to 0)^b^
ABG (mean, SD) dB^c^	34 (10)	31 (8)	2 (–1 to 6)^b^
Maximum hearing level (mean (SD) dB)^d^	98 (12)	98 (11)	0 (–4 to 4)^b^
Maximum hearing level (mean (SD) %)^d^	100 (1)	99 (3)	1 (0 to 2)^b^
Hearing level at 65dB (mean (SD) dB)	29 (30)	29 (30)	0 (–11 to 11)^b^
Intraoperative characteristics			
Operated side (left:right)	22:32	26:29	^a^
Revision surgery (n (%))	11 (20)	11 (20)	^a^
Type of previous surgery (n (%))			
Stapedotomy	8 (73)	6 (55)	^a^
Stapedectomy	0 (0)	3 (27)	
Other (middle ear inspection/mobilization)	3 (27)	2 (18)	
Type of surgery (n (%))			
Stapedotomy	50 (93)	53 (96)	^a^
Stapedectomy	1 (2)	0 (0)	
Other (middle ear inspection/mobilization)	3 (6)	2 (4)	
Type of fenestration (n (%))			
KTP Laser	44 (81)	46 (84)	^a^
Micropick	2 (4)	3 (5)	
Microdrill	1 (2)	1 (2)	
None	7 (13)	5 (9)	
Type of piston (n (%))			
Fluoroplastic Teflon (large) loop piston	42 (78)	44 (80)	^a^
Titanium K-piston	10 (19)	9 (16)	
Angular titanium piston	1 (2)	0 (0)	
None	1 (2)	1 (2)	
Diameter of piston (n (%))			
0.3 mm	13 (25)	4 (7)	^a^
0.4 mm	34 (64)	43 (80)	
0.6 mm	2 (4)	2 (4)	
0.8 mm	3 (6)	4 (7)	
Missing	1 (2)	0 (0)	
Duration of surgery (mean [SD] in minutes)	59 (15)	59 (14)	0 (–5 to 6)^b^
Time of surgery (mean [SD] time)	10:08 (1:38)	10:11 (1:46)	–0:03 (–0:42 to 0:36)^b^

^a^Fisher’s exact test.

^b^Independent-samples Student’s *t*-test.

^c^Calculated using BC and/or AC thresholds at 0.5, 1, 2, and 4 kHz.

^d^dB with maximum percentage of speech recognition in quiet.

None of the baseline characteristics were statistically significant.

ABG = air-bone gap; AC = air conduction; BC = bone conduction; CI = confidence interval; KTP = potassium titanyl phosphate; n = number of cases; SD = standard deviation.

### Audiometric Measurements

**FIG. 3.**
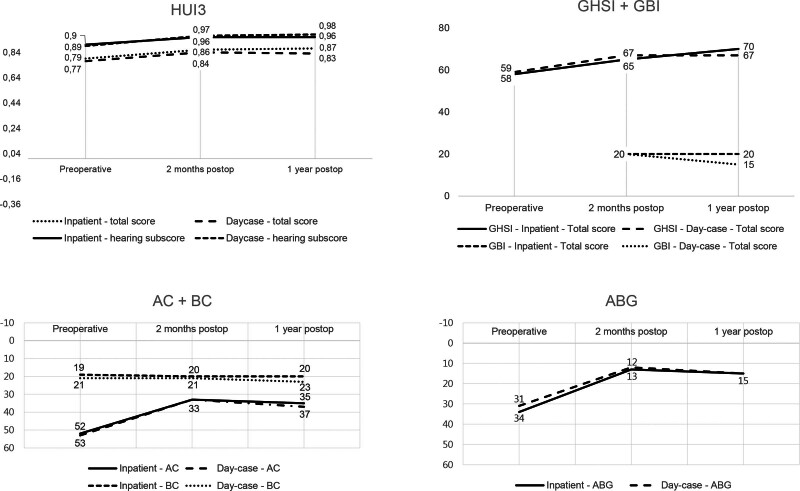
Outcomes. ABG indicates air-bone gap; AC, air-conduction; BC, bone-conduction; GBI, Glasgow Benefit Inventory; GHSI, Glasgow Health Status Inventory; HUI3, Health Utilities Index—Mark 3.

The mean AC did not differ at 2 months and 1 year postoperatively (0 dB, 95% CI, –4 to 5 and –2 dB, 95% CI, –8 to 4 respectively) (Table 2; Fig. 3). Neither the mean BC, nor the mean ABG differed at 2 months and 1 year postoperatively. There was no difference in ABG closure to 10 dB or less between the inpatient and day-case group at 2-month and 1-year follow-up. The difference in ABG closure to 20 dB or less was also not significant at both follow-up moments. The mean difference in maximum hearing level was 0 dB (95% CI, –5 to 5) and –3% (95% CI, –8 to 2) at 2-month follow-up and –3 dB (95% CI, –10 to 4) and –3% (95% CI, –8 to 2) at 1-year follow-up. The mean difference in speech recognition scores at 2-month follow-up was 1% (95% CI, –9 to 12) and at 1-year follow-up 5% (95% CI, –6 to 17).

**TABLE 2.** Postoperative audiometric results

	Inpatient (n = 54)		Day case (n = 55)		Difference (95% CI)	
	2 months	1 year	2 months	1 year	2 months	1 year
AC (mean (SD)) dB^a^	33 (14)	35 (16)	33 (11)	37 (15)	0 (–4 to 5)^b^	–2 (–8 to 4)^c^
BC (mean (SD)) dB^a^	20 (14)	20 (9)	21 (8)	23 (8)	–1 (–5 to 4)^b^	–2 (–6 to 2)^b^
ABG (mean (SD)) dB^a^	13 (12)	15 (9)	12 (7)	15 (10)	1 (–3 to 4)^c^	0 (–4 to 4)^c^
ABG closure ≤10 dB (n = yes (%))^a^	26 (48)	16 (30)	26 (47)	22 (40)	0% (–18 to 19)^d^	–10% (–28 to 8)^d^
ABG closure ≤20 dB (n = yes (%))^a^	47 (87)	44 (81)	50 (91)	42 (76)	–3% (–16 to 8)^d^	5% (–10 to 20)^d^
Maximum hearing level (mean (SD)) dB^e^	80 (12)	79 (13)	80 (13)	82 (15)	0 (–5 to 5)^b^	–3 (–10 to 4)^c^
Maximum hearing level (mean (SD))%^e^	96 (19)	95 (16)	99 (3)	98 (2)	–3 (–8 to 2)^c^	–3 (–8 to 2)^c^
Speech recognition(mean (SD)) %^f^	78 (26)	77 (26)	76 (28)	72 (32)	1 (–9 to 12)^b^	5 (–6 to 17)^c^

^a^Calculated using BC and AC thresholds at 0.5, 1, 2, and 4 kHz.

^b^Independent-samples Student’s *t*-test.

^c^Independent-samples Mann-Whitney *U* test.

^d^Fisher’s exact test. None of the differences were statistically significant (*P* < 0.05).

^e^dB with maximum percentage of speech recognition in quiet.

^f^Percentages of speech recognition at 65 dB SPL in quiet.

ABG indicates air-bone gap; AC, air conduction; BC, bone conduction; CI, confidence interval; n, number of patients; SD, standard deviation; SPL, sound pressure level.

### Quality of Life

The disease-specific quality of life (QoL) measured by the GHSI and the overall QoL measured by the HUI3 did not show a statistically significant difference between the inpatient and the day-case group (Table 3; Fig. 3). The change in health status measured by the GBI as a result of the stapes surgery also did not differ significantly between both groups.

**TABLE 3.** Questionnaires

	Inpatient			Day-case			Difference (95% CI)		
	Preoperative	3 months	1 year	Preoperative	3 months	1 year	Preoperative	3 months	1 year
GHSI (mean (SD))	n = 53	n = 46	n = 40	n = 54	n = 49	n = 38			
*Total score*	58 (12)	65 (16)	70 (13)	59 (12)	67 (14)	67 (14)	–1 (–6 to 4)^a^	–2 (–8 to 4)^a^	3 (–3 to 9)^a^
*General subscore*	52 (16)	64 (21)	70 (17)	54 (16)	67 (18)	67 (18)	–2 (–8 to 4)^a^	–3 (–11 to 5)^a^	3 (–5 to 11)^a^
*Social support subscore*	77 (16)	78 (16)	76 (19)	77 (15)	76 (17)	73 (18)	0 (–6 to 6)^b^	2 (–5 to 9)^a^	3 (–5 to 11)^a^
*Physical health subscore*	62 (21)	60 (20)	60 (21)	62 (18)	62 (19)	58 (17)	0 (–8 to 8)^a^	–2 (–9 to 6)^a^	2 (–7 to 10)^a^
GBI (mean (SD))		n = 48	n = 41		n = 49	n = 37			
*Total score*		20 (23)	20 (17)		17 (13)	15 (17)		3 (–5 to 12)^a^	5 (–3 to 12)^a^
*General subscore*		29 (31)	29 (22)		24 (21)	23 (24)		4 (–7 to 16)^b^	6 (–4 to 16)^a^
*Social support subscore*		6 (21)	3 (9)		3 (12)	1 (7)		3 (–5 to 11)^b^	2 (–2 to 5)^b^
*Physical health subscore*		–1 (16)	0 (11)		–1 (6)	–1 (13)		0 (–6 to 6)^b^	2 (–4 to 8)^b^
HUI3 (mean (SD))	n = 52	n = 45	n = 43	n = 55	n = 48	n = 41			
*Multi attribute score*	0.79 (0.19)	0.86 (0.20)	0.87 (0.17)	0.77 (0.19)	0.84 (0.17)	0.83 (0.22)	0.02 (–0.05 to 0.09)^a^^b^	0.02 (–0.06 to 0.09)^b^	0.05 (–0.04 to 0.13)^b^
*Hearing multi utility score*	0.90 (0.10)	0.96 (0.09)	0.96 (0.08)	0.89 (0.10)	0.97 (0.07)	0.98 (0.07)	0.01 (–0.03 to 0.05)^b^	0.00 (–0.04 to 0.03)^b^	–0.01 (–0.05 to 0.02)^b^
**Tinnitus**									
THI (mean (SD))	n = 31	n = 21	n = 21	n = 36	n = 28	n = 23			
*Total score*	11 (14)	15 (13)	4 (3)	23 (22)	22 (18)	18 (20)	–**12** (–**20 to** –**4**)^b^	–7 (–16 to 2)^b^	–8 (–17 to 2)^b^
*Emotional response*	2 (3)	3 (4)	2 (2)	5 (6)	5 (6)	4 (6)	–3 (–5 to 0)^b^	–2 (–5 to 2)^b^	–2 (–5 to 0)^b^
*Functional response*	6 (8)	7 (7)	6 (4)	13 (11)	12 (9)	11 (11)	–**6** (–**11 to** –**2**)^b^	–**5** (–**10 to 0**)^b^	–5 (–19 to 0)^b^
*Catastrophic response*	3 (3)	4 (3)	3 (3)	6 (4)	5 (4)	4 (4)	–**2** (–**4 to 0**)^b^	–1 (–3 to 1)^b^	0 (–2 to 2)^b^
THI; severity (n (%))	n = 31	n = 21	n = 21	n = 36	n = 28	n = 23			
*Slight handicap*	22 (71)	15 (71)	18 (86)	20 (56)	16 (57)	15 (65)	^c^	^c^	^c^
*Mild handicap*	7 (23)	5 (24)	3 (14)	10 (28)	6 (21)	4 (17)			
*Moderate handicap*	2 (6)	1 (5)	0 (0)	3 (8)	5 (18)	2 (9)			
*Severe handicap*	0 (0)	0 (0)	0 (0)	3 (8)	1 (4)	2 (9)			
TQ (mean (SD))	n = 30	n = 23	n = 21	n = 34	n = 28	n = 21			
*Total score*	14 (12)	16 (10)	9 (6)	22 (15)	19 (11)	18 (12)	–**8** (–**14 to** –**1**)^b^	–3 (–10 to 4)^b^	–**8** (–**14 to** –**2**)^b^
*Emotional distress*	4 (4)	5 (4)	3 (3)	7 (7)	6 (5)	5 (4)	–**4** (–**6 to** –**1**)^b^	–1 (–4 to 2)^b^	–2 (–5 to 0)^b^
*Auditory perceptual difficulties*	4 (4)	4 (4)	2 (2)	4 (3)	3 (2)	3 (3)	–1 (–2 to 1)^b^	1 (–1 to 2)^b^	0 (–2 to 1)^b^
*Intrusiveness*	5 (3)	4 (3)	3 (2)	6 (4)	6 (3)	5 (3)	–1 (–3 to 0)^b^	–2 (–4 to 1)^b^	–**2** (–**4 to 0**)^b^
*Sleep disturbances*	1 (1)	1 (2)	1 (1)	2 (2)	2 (2)	2 (2)	–**1** (–**2 to 0**)^b^	–1 (–2 to 0)^b^	–**2** (–**3 to 0**)^b^
*Somatic complaints*	1 (1)	1 (2)	0 (1)	1 (2)	1 (1)	1 (2)	–1 (–2 to 0)^b^	–1 (–1 to 1)^b^	–1 (–2 to 0)^b^
UBQ Tinnitus	n = 28	n = 22	n = 20	n = 36	n = 30	n = 23			
VAS (mean (SD))	4 (3)	4 (2)	4 (2)	5 (3)	4 (2)	5 (2)	–1 (–2 to 1)^a^	–1 (–2 to 0)^b^	–1 (–2 to 1)^a^
**Vertigo**									
DHI (mean (SD))	n = 10	n = 6	n = 6	n = 6	n = 7	n = 9			
*Total score*	11 (9)	39 (30)	12 (8)	21 (13)	17 (17)	16 (15)	–10 (–23 to 2)^b^	22 (–4 to 47)^b^	–4 (–17 to 9)^b^
*Physical subscore*	6 (5)	15 (7)	6 (6)	9 (7)	7 (7)	9 (6)	–3 (–9 to 3)^b^	7 (–1 to 15)^b^	–3 (–9 to 4)^b^
*Functional subscore*	3 (4)	12 (12)	2 (2)	8 (4)	5 (5)	5 (6)	–5 (–10 to 1)^b^	7 (–3 to 17)^b^	–3 (–8 to 3)^b^
*Emotional subscore*	2 (2)	12 (11)	3 (5)	4 (5)	5 (7)	2 (4)	–2 (–6 to 1)^b^	7 (–2 to 17)^b^	1 (–3 to 5)^b^
DHI; severity (n (%))	n = 10	n = 6	n = 6	n = 6	n = 7	n = 9			
*No handicap*	8 (80)	1 (17)	3 (50)	3 (50)	4 (57)	3 (33)	^c^	^c^	^c^
*Mild handicap*	2 (20)	3 (50)	3 (50)	1 (17)	2 (29)	5 (56)			
*Moderate handicap*	0 (0)	1 (17)	0 (0)	2 (33)	1 (14)	1 (11)			
*Severe handicap*	0 (0)	1 (17)	0 (0)	0 (0)	0 (0)	0 (0)			
UBQ Vertigo	n = 10	n = 6	n = 6	n = 5	n = 7	n = 9			
VAS (mean (SD))	2 (2)	5 (3)	2 (1)	2 (2)	2 (2)	3 (2)	–1 (–3 to 2)^b^	3 (0 to 6)^b^	–1 (–2 to 1)^b^

Differences printed in bold were statistically significant (*P* < 0.05).

^a^Independent-samples Student’s *t*-test.

^b^Independent-samples Mann-Whitney *U* test.

^c^Fisher’s exact test.

CI indicates confidence interval; DHI, Dizziness Handicap Inventory; GBI, Glasgow Benefit Inventory; GHSI, Glasgow Health Status Inventory; HUI3, Health Utility Index 3; n, number of patients; SD, standard deviation; TBQV, Utrecht Burden Questionnaire Vertigo; THI, Tinnitus Handicap Inventory; TQ, Tinnitus Questionnaire; UBQT, Utrecht Burden Questionnaire Tinnitus.

### Patient Satisfaction

The overall mean patient satisfaction score of the first postoperative night on a scale of 0 (very easy) to 10 (very difficult) was 4.8 (SD 2.8) in both groups (Appendix 3, http://links.lww.com/ONO/A8). The only statistically significant between-group difference was that 44% of the patients allocated to the inpatient group felt less anxious due to the group, they were allocated to, compared with 13% of the day-case group patients. However, when the question was asked if patients were more anxious because the surgery was planned in an inpatient or day-case setting, there was no significant difference (6%, 95% CI, –6 to 21). Of the patients allocated to the day-case group, 77% would not have preferred to have spent the night in the hospital after surgery and 73% would undergo future surgery in a day-case setting again.

### Postoperative Complications, Tinnitus, and Vertigo

Intraoperative complications were all minor and included a fractured footplate (n = 2 inpatient; n = 1 day-case) and a floating footplate (n = 2 inpatient; n = 1 day-case) (Table 4). Direct postoperative complications were all classified as minor and included nausea (n = 5 inpatient; n = 2 day-case), PONV (n = 5 inpatient; n = 4 day-case) with dizziness (n = 3 inpatient; n = 1 day-case) or vertigo (n = 0 inpatient; n = 3 day-case), dizziness (n = 6 inpatient; n = 8 day-case), vertigo (n = 2 inpatient; n = 2 day-case), and pain (n = 1 inpatient; n = 0 day-case). Three patients in the inpatient group stayed one extra night postoperatively due to PONV, dizziness, or vertigo. Early postoperative complications were all minor and included tinnitus (n = 23 inpatient; n = 28 day-case), vertigo (n = 6 inpatient; n = 6 day-case), infection or otitis externa (n = 2 inpatient; n = 2 day-case), and impaired wound healing (n = 3 inpatient; n = 2 day-case). Over the follow-up period of 1 year, six patients required revision surgery (n = 1 inpatient; n = 5 day-case), one patient for a tympanoplasty and five patients for middle ear inspection due to (persistent) conductive hearing loss. Tinnitus (n = 21 inpatient; n = 21 day-case), vertigo (n = 6 inpatient; n = 8 day-case), SNHL (n = 2 inpatient; n = 5 day-case), and a persistent altered taste (n = 3 inpatient; n = 1 day-case) were the late postoperative complications. Only SNHL was classified as a major complication. None of the complication rates were statistically significantly different between both groups.

**TABLE 4.** Complications

	Inpatient (n = 54)	Day-case (n = 55)
Intraoperative complications (n (%))		
Fractured footplate	2 (4)	1 (2)
Floating footplate	2 (4)	1 (2)
Direct postoperative complications (n (%))		
None	32 (59)	35 (64)
Nausea	5 (9)	2 (4)
PONV	5 (9)	4 (7)
Dizziness	6 (11)	8 (15)
Vertigo	2 (4)	2 (4)
PONV and dizziness	3 (6)	1 (2)
PONV and vertigo	0 (0)	3 (5)
Pain	1 (2)	0 (0)
Early postoperative complications (n (%)), <1 month		
None	21 (39)	14 (25)
Tinnitus	23 (43)	28 (51)
Vertigo	6 (11)	6 (11)
Infection/otitis externa	2 (4)	2 (4)
Impaired wound healing	3 (6)	2 (4)
Late postoperative complications (n (%)), 1 year		
None	17 (31)	12 (13)
Tinnitus	21 (39)	21 (38)
Vertigo	6 (11)	8 (15)
SNHL	2 (4)	5 (9)
Revision surgery	1 (2)	5 (9)
Persistent altered taste	3 (6)	1 (2)

None of the differences were statistically significant (*P* < 0.05) using the Fisher’s Exact Test (2-sided).

n indicates number of patients; PONV, postoperative nausea and vomiting; SNHL, sensorineural hearing loss.

#### Tinnitus

The THI and TQ showed a higher preoperative tinnitus burden in the day-case group (mean difference THI –12, 95% CI, –20 to –4 and mean difference TQ –8, 95% CI, –14 to –1) (Table 3). At 3-month follow-up, only the functional subscore of the THI was significantly higher in the day-case group (mean difference –5, 95% CI, –10 to 0). At 1-year follow-up, the total score and the intrusiveness and sleep disturbance subscores of the TQ were higher in the day-case group (–8, 95% CI, –14 to –2; –2, 95% CI, –4 to 0 and –2, 95% CI, –3 to 0 points, respectively).

#### Vertigo

There were no differences between the inpatient and day-case group measured by the DHI and UBQV questionnaires (Table 3).

### Crossover and Readmission

Of the patients allocated to the day-case group, there was a crossover rate of 38% (20 patients). These patients required a one-night (n = 16), two-night (n = 2), three-night (n = 1), or four-night (n = 1) admission to the ward. Reasons therefor were PONV (n = 5; 25%), vertigo (n = 4; 20%), dizziness (n = 8; 40%), at request of the surgeon (n = 1; 5%), or at request of the patient (n = 2; 10%). All but one of our crossover patients (day-case to inpatient care) were out of surgery by 14:00 pm. Within the inpatient group, there was a crossover rate of 12% (6 patients), all at request of the patient. No patients required readmission after initial discharge.

## DISCUSSION

This study allows for a comparison of hearing outcomes, QoL, patient satisfaction, postoperative complications, and crossover and readmission rates between inpatient and day-case stapes surgery. We found equal postoperative AC scores between both groups, and there were no statistically significant between-group differences in the other audiometric measurements. There were no statistically significant differences between the inpatient and day-case group regarding the overall QoL (HUI3), disease-specific QoL (GHSI), change in postoperative health status (GBI), and postoperative complication rates. Preoperatively, both the THI and TQ questionnaires showed a statistically significant, but clinically irrelevant, difference in tinnitus burden in favor of the inpatient group. However, postoperatively most of these (sub)scores did not differ anymore, except for the THI functional response subscore at 3 months and the TQ total score and intrusiveness and sleep disturbance subscores at 1-year follow-up. Given the minimal differences between these scores, they were seen as clinically irrelevant.

When defining the feasibility of day-case stapes surgery in terms of crossover rate, 38% of our day-case patients ended up staying at least one night postoperatively. Our admission rate after day-case surgery is substantially higher than previously reported admission rates. In our previous systematic review, we found 15 studies reporting admission rates of 0% to 13% following day-case stapes surgery (19). Only three of our crossover patients from day-case to inpatient care were due to nonmedical reasons: at request of the surgeon without specific reason or at request of the patient due to social reasons. Our number of patients with PONV and dizziness/vertigo is comparable to those reported in other articles and are described to be the main causes of failure for a day-case approach. Lazard et al found that 60% of the crossover cases following day-case stapes surgery were due to nausea, vomiting, and dizziness (50). In our review, we found that 53% of the patients requiring admission after stapes surgery suffered from postoperative vertigo/dizziness with or without nausea and 17% suffered from PONV. Other reasons for failure of a day-case approach were social reasons (17%), late-scheduled surgery (6%), or asthenia (3%) (19). Direct postoperative vertigo and dizziness after stapes surgery might be a result of the intraoperative stimulation of the vestibular labyrinth or as an effect of the general anesthesia. The possible effect of general anesthesia on these symptoms has been hypothesized by Dornhoffer et al, who performed day-case major otologic surgery under local anesthesia in 112 patients with only one patient requiring postoperative admission due to vertigo after revision stapes surgery (51). An increased risk of PONV has been associated with a history of PONV or motion sickness and the use of narcotics (32,52,53). Therefore, a potential way to increase the feasibility of day-case stapes surgery is to reduce postoperative nausea and dizziness/vertigo by using pre- and postoperative antiemetic drugs, less and shorter-acting intraoperative anesthetics and avoiding the use of opioids (31,32). The majority of our study population received intra- or postoperative antiemetic drugs (n = 49 inpatient; n = 46 day-case), received the same combination of anesthetics and were intubated for surgery. Therefore, it is not possible to determine if these factors were a potential cause for failure of a day-case approach. We did find that the use of morphine or opioids were significantly higher in the crossover (day-case to inpatient) group, whereby 95% received morphine or opioids compared to 71% of the day-case patients that were discharged the day of surgery.

Other ways to increase the feasibility of day-case surgery is careful preoperative patient selection whereby both social and medical factors need to be considered. Social factors include the patients’ home situation with easy access to a telephone and someone to take care of the patient for at least the first postoperative night. The most important medical factor is a health status allowing general anesthesia in a day-case setting (32). Besides this, the surgical planning is something to consider, leaving enough postoperative recovery time before discharge of a day-case patient. A final consideration is the perioperative admission to a day-case unit instead of the inpatient ward, which seems to enhance a day-case mindset of both the patient and the involved personnel (surgeon, anesthesiologist and paramedical team) (50).

Besides the feasibility, we assessed the desirability of a day-case approach of our study population. An interesting finding is that 23% of the inpatient patients were less anxious due to the fact that the surgery was planned in an inpatient setting compared with only 7% of the day-case patients being less anxious due to the fact that the surgery was planned in day-case. However, only 6% of the day-case patients were more anxious because they were planned in a day-case setting. Seventy-three percent of our day-case patients would undergo the surgery in a day-case setting again. Ralli et al evaluated the patient satisfaction with stapes surgery as a day-case approach and found that 96% would repeat the surgery as a day-case procedure (54). Moreover, six of our patients allocated to the inpatient group were discharged on the day of surgery at their own request.

In interpreting these findings, the following considerations need to be considered. A limitation of this trial is that a preoperative selection of our patients has been made including only patients in good health and with quick access to communication and transportation. Second, our protocol reads that we planned a sensitivity analysis using data acquired from patients that opted not to be included in the study. Unfortunately, only four patients were willing to fulfill the questionnaires without participating in the randomization process. Therefore, we decided not to perform a sensitivity analysis and as a result, the generalizability of this study was not assessed. A final limitation concerning the used outcome measures is that only the translated Dutch TQ questionnaire has been validated. Also, the TQ, THI and DHI questionnaire are not validated for assessment of treatment outcome. However, tinnitus and vertigo were not a primary outcome in this study but assessed as a complication.

## CONCLUSION

When taking the postoperative hearing outcomes, the overall and disease-specific QoL, patient satisfaction, and postoperative complication rates into account, a day-case approach of stapes surgery showed no clinically relevant differences compared with an inpatient surgical approach. Deliberate patient selection, surgical planning, and changing the mindset toward a day-case approach of both patient and the surgical team, will increase the acceptance and the feasibility of day-case stapes surgery.

## FUNDING SOURCES

None declared.

## CONFLICT OF INTEREST

None declared.

## DATA AVAILABILITY

The datasets generated during and/or analyzed during the current study are not publicly available, but are available from the corresponding author on reasonable request.

## Supplementary Material


