# Supplementary material for: Hearing Results, Quality of Life, Patient Satisfaction, and Postoperative Complications of Day-case Versus Inpatient Stapes Surgery for Otosclerosis in Adults: A Randomized Controlled Trial
Source: Otol Neurotol Open. 2022 Oct 27;2(4):e019. doi: 10.1097/ONO.0000000000000019 (PMC10950158; doi:10.1097/ONO.0000000000000019)
Supplement: Supplementary file 2 [file ono-2-e019-s002.pdf]

Utrecht patient satisfaction survey

Day-case stapes surgery
Day-case surgery means that you have been admitted one day before or the day of surgery and have been discharged the day of the surgery.

Q1.	Did you feel more anxious because the surgery was planned in a day-case setting?	Yes	No
Q2.	Did you feel less anxious because the surgery was planned in a day-case setting?	Yes	No
Q3.	Did you find it pleasant that you did not have to spend the night in the hospital after the surgery?	Yes	No
Q4.	If you would have the choice: would you undergo the surgery in day-case setting again next time?	Yes	No
Q5.	Would you have preferred to have spent the night in the hospital after the surgery?	Yes	No
Q6.	Were you content with the hospital admittance in general?	Yes	No
Q7.	How easy or difficult was the first night after the operation on a scale from 0 to 10 (0 is very easy and 10 is as difficult as possible)? 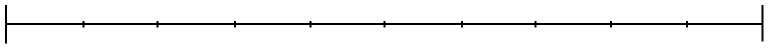 0 100		

Utrecht patient satisfaction survey

Inpatient stapes surgery
Inpatient surgery means that you have been admitted one day before or the day of surgery followed by one-day hospital admittance.

Q1.	Did you feel more anxious because the surgery was planned in an inpatient setting?	Yes	No
Q2.	Did you feel less anxious because the surgery was planned in an inpatient setting?	Yes	No
Q3.	Did you find it pleasant that you had to spend the night in the hospital after the surgery?	Yes	No
Q4.	If you would have the choice: would you undergo the surgery in an inpatient setting again next time?	Yes	No
Q5.	Would you have preferred to have spent the night at home after the surgery?	Yes	No
Q6.	Were you content with the hospital admittance in general?	Yes	No
Q7.	How easy or difficult was the first night after the operation on a scale from 0 to 10 (0 is very easy and 10 is as difficult as possible)?<br>0 —————————— 100		
