# Supplementary material for: Hearing Results, Quality of Life, Patient Satisfaction, and Postoperative Complications of Day-case Versus Inpatient Stapes Surgery for Otosclerosis in Adults: A Randomized Controlled Trial
Source: Otol Neurotol Open. 2022 Oct 27;2(4):e019. doi: 10.1097/ONO.0000000000000019 (PMC10950158; doi:10.1097/ONO.0000000000000019)
Supplement: Supplementary file 3 [file ono-2-e019-s003.pdf]

Appendix 3. Utrecht Patient Satisfaction Survey

	**Inpatient (n=52)**	**Day-case (n=52)**	**Difference (95% CI)**
Q1 (Did you feel more anxious because the surgery was planned in an inpatient/a day-case setting?) (n (%))<br>*Yes*	6 (12)	3 (6)	6% (-6 to 21)*
Q2 (Did you feel less anxious because the surgery was planned in an inpatient/a day-case setting?) (n (%))<br>*Yes*	23 (44)	7 (13)	**31% (13 to 46)***
Q3 (Did you find it pleasant that you had/did not have to spend the night in the hospital after the surgery?) (n (%))<br>*Yes*	44 (85)	42 (81)	4% (-11 to 18)*
Q4 (If you would have the choice: would you undergo the surgery in an inpatient/day-case setting again next time?) (n (%))<br>*Yes*	41 (79)	38 (73)	6% (-11 to 22)*
Q5 (Would you have preferred to have spent the night at home/in the hospital after the surgery?) (n (%))<br>*Yes*	10 (19)	12 (23)	-4% (-19 to 12)*
Q6 (Were you content with the hospital admittance in general?) (n (%))<br>*Yes*	51 (98)	50 (96)	2% (-6 to 10)*
Q7 (How easy or difficult was the first night after the operation on a scale from 0 to 10?) (mean (SD))	4.8 (2.8)	4.8 (2.8)	0 (-1 to 1)^

* Fisher's exact test (2-sided); ^ Independent-samples Student's *t*-test. Differences printed in bold were statistically significant ($p<0.05$).
